# Analyses of the probiotic property and stress resistance-related genes of *Lactococcus lactis* subsp. *lactis* NCDO 2118 through comparative genomics and *in vitro* assays

**DOI:** 10.1371/journal.pone.0175116

**Published:** 2017-04-06

**Authors:** Letícia C. Oliveira, Tessália D. L. Saraiva, Wanderson M. Silva, Ulisses P. Pereira, Bruno C. Campos, Leandro J. Benevides, Flávia S. Rocha, Henrique C. P. Figueiredo, Vasco Azevedo, Siomar C. Soares

**Affiliations:** 1 Laboratory of Cellular and Molecular Genetics, Institute of Biological Sciences, Federal University of Minas Gerais, Belo Horizonte—MG, Brazil; 2 Department of Preventive Veterinary Medicine, State University of Londrina, Londrina—PR, Brazil; 3 Official Laboratory of Fisheries Ministry—Veterinary School, Federal University of Minas Gerais, Belo Horizonte, MG, Brazil; 4 Department of Microbiology, Immunology and Parasitology, Institute of Biological and Natural Sciences, Federal University of Triângulo Mineiro, Uberaba—MG, Brazil; University of Torino, ITALY

## Abstract

*Lactococcus lactis* subsp. *lactis* NCDO 2118 was recently reported to alleviate colitis symptoms via its anti-inflammatory and immunomodulatory activities, which are exerted by exported proteins that are not produced by *L*. *lactis* subsp. *lactis* IL1403. Here, we used *in vitro* and *in silico* approaches to characterize the genomic structure, the safety aspects, and the immunomodulatory activity of this strain. Through comparative genomics, we identified genomic islands, phage regions, bile salt and acid stress resistance genes, bacteriocins, adhesion-related and antibiotic resistance genes, and genes encoding proteins that are putatively secreted, expressed *in vitro* and absent from IL1403. The high degree of similarity between all *Lactococcus* suggests that the Symbiotic Islands commonly shared by both NCDO 2118 and KF147 may be responsible for their close relationship and their adaptation to plants. The predicted bacteriocins may play an important role against the invasion of competing strains. The genes related to the acid and bile salt stresses may play important roles in gastrointestinal tract survival, whereas the adhesion proteins are important for persistence in the gut, culminating in the competitive exclusion of other bacteria. Finally, the five secreted and expressed proteins may be important targets for studies of new anti-inflammatory and immunomodulatory proteins. Altogether, the analyses performed here highlight the potential use of this strain as a target for the future development of probiotic foods.

## Introduction

The genus *Lactococcus* is part of the lactic acid bacteria (LAB), one of the most biotechnologically important groups of bacteria, which is composed of *Lactococcus*, *Streptococcus*, *Lactobacillus*, *Weissella* and others [1]. LAB species share in common the ability to convert sugar (mainly glucose) into lactic acid through specific metabolic pathways. Additionally, these species are facultative anaerobic, catalase negative and non-motile. Moreover, there is a close phylogenetic relationship between the bacteria of this group [2].

Many LAB species are biotechnologically important due to their safety aspects, achieved because they have been used for years in the preservation and maintenance of food [3]. Previous studies highlight the importance of genome sequencing in the discovery of new features related to LAB: genes coding for proteolytic enzymes (which participate in cheese maturation) in *Lactobacillus helveticus* [4], identification of citrate catabolic pathways in *Lactobacillus casei* [5], and genes responsible for decarboxylation of alpha-keto acid branched chain in *Lactococcus lactis* [6; 7].

Genome sequencing studies have also helped in the elucidation of probiotic effects exerted by LAB. For instance, in *Lactobacillus reuteri*, genome analyses have focused on the capacity to adapt to nutrient availability and environmental conditions of the GI tract, the adhesion mechanisms, the production of antimicrobial compounds, and the mechanisms of immunomodulation, such as the synthesis of pro-inflammatory extracellular polymeric substances (EPS compounds) [8]. Moreover, *Lactobacillus rhamnosus* and *L*. *casei* strains isolated from marketed probiotic products were compared with the well-studied *L*. *rhamnosus* GG and *L*. *casei* BL23, mainly focusing on pilus gene clusters and metabolic pathways analyses [9]. Interestingly, a new adhesion-associated protein, *cwaA*, was identified through genome sequencing and comparative genomics analyses of *Lactobacillus plantarum* NL42. The expression of cwaA in *L*. *lactis* has significantly increased its autoaggregation, hydrophobicity and exclusion ability, where the mutant strain was able to inhibit the adhesion of *Staphylococcus aureus* and *Escherichia coli* to HT-29 cells [10]. Another study illustrated the mechanisms by which *Lactobacillus* species from the intestinal niche have adapted to the gastrointestinal tract (GIT) by acquiring traits, such as stress tolerance, carbohydrate absorption, adhesion to epithelial cells and mucus [11].

Additionally, many species of this group are important for their probiotic effects, such as the genus *Lactobacillus*, which is used in the production of the fermented milk Yakult [12], and *Bifidobacteria*, widely known for their beneficial effects on the host intestinal tract [13]. However, although several works highlight the probiotic effects of LAB, most focus on *Lactobacillus* and *Bifidobacterium* species [14], whereas few studies report the beneficial effects of *L*. *lactis* strains. For instance, *Lactococcus lactis* subsp. *cremoris* FC has an important anti-inflammatory activity [15]. The probiotic properties of *L*. *lactis* subsp. *cremoris* IBB477 have attracted attention due to their adhesion mechanisms and survival in the intestinal environment [16; 17]. Additionally, it was recently demonstrated, through the evaluation of three *L*. *lactis* strains *in vitro*, that *Lactococcus lactis* subsp. *lactis* NCDO 2118 has anti-inflammatory and immunomodulatory activity that can alleviate colitis symptoms [18]. This strain was described as a gamma-aminobutyric acid (GABA) producer [19]. It has been extensively used for heterologous expression [20], and its probiotic effect is associated with exported proteins [18].

Here, we use comparative genomics and *in silico* analyses to provide insights into the probiotic nature of *L*. *lactis* NCDO 2118. The criteria for screening LAB strains before their use as probiotics include assessing functional features, such as the ability to resist environmental conditions found in the digestive tract (low gastric pH and bile salts) and the ability to antagonize or competitively exclude pathogens, which is achieved by secreting antimicrobial substances or competing for nutrients and epithelial adhesion sites. LAB produce different antimicrobial components, such as organic acids, hydrogen peroxide, carbon peroxide, diacetyl, low molecular weight antimicrobial substances, bacteriocins and adhesion inhibitors. The adhesiveness of LAB may involve passive forces, electrostatic interactions, hydrophobic steric forces, lipoteichoic acids, and lectins [21]. The hydrophobic nature of the outermost surface of microorganisms facilitates the adhesion of bacteria to the host epithelium, thereby conferring competitive advantages during the colonization of the GIT [22]. The antimicrobial susceptibility of intestinal microorganisms is an important criterion for the selection of probiotic strains, mainly due to the potential transfer of those genes to pathogenic or commensal bacteria that inhabit the GIT [23]. In the following sections, we present comparative genomic analyses of *L*. *lactis* NCDO 2118 and other *Lactococcus* species and predict genes that putatively code for acid stress resistance proteins, bacteriocins, adhesins and exported proteins.

## Results

### General features, phylogenomics and synteny analyses

The general genomic features of all genomes used in this work are summarized in Table 1.

**Table 1:** Complete genomes and genomic features of *Lactococcus* species and *Streptococcus thermophilus* used in genomic comparisons.

Strain	Size (bp)	GC%	Genes	Proteins	Source	Accession Number	Plasmids	Pseudogenes	Reference
***Lactococcus lactis* subsp. *lactis* NCDO 2118**	2,554,693	34,86	2,471	2,386	Frozen peas	CP009054	1	52	[24]
***Lactococcus lactis* subsp. *lactis* IL1403**	2,365,589	35,30	2,406	2,277	Dairy starter	AE005176	-	45	[3]
***Lactococcus lactis* subsp. *lactis* KF147**	2,598,144	34,86	2,662	2,473	Mung Bean	CP001834	1	93	[25]
***Lactococcus lactis* subsp. *lactis* KLDS 40325**	2,589,250	35,39	2,732	2,593	Fermented milk	CP006766	1	56	[26]
***Lactococcus lactis* subsp. *lactis* IO-1**	2,421,471	35,10	2,318	2,224	Water (drain pit of a kitchen sink)	AP012281	-	-	[27]
***Lactococcus lactis* subsp. *lactis* CV56**	2,399,458	35,09	2,549	2,408	Healthy woman’s vagina	CP002365	5	51	[28]
***Lactococcus lactis* subsp. *lactis* S0**	2,488,699	35,20	2,482	2,311	Fresh raw milk	CP010050	-	88	Unpublished
***Lactococcus lactis* subsp. *lactis* AI06**	2,398,091	35,04	2,320	2,178	Açaí palm	CP009472	-	61	[29]
***Lactococcus lactis* subsp. *cremoris* A76**	2,452,616	35,88	2,845	2,769	Dairy starter	CP003132	4	-	[30]
***Lactococcus lactis* subsp. *cremoris* KW2**	2,427,048	35,70	2,353	2,268	Fermented corn	CP004884	-	1	[31]
***Lactococcus lactis* subsp. *cremoris* MG1363**	2,529,478	35,70	2,597	2,434	Dairy starter	AM406671	-	82	[32]
***Lactococcus lactis* subsp. *cremoris* NZ9000**	2,530,294	35,70	2,594	2,510	Dairy starter	CP002094	-	-	[33]
***Lactococcus lactis* subsp. *cremoris* SK11**	2,438,589	35,82	2,739	2,501	Dairy starter	CP000425	5	144	[34]
***Lactococcus lactis* subsp. *cremoris* UC5099**	2,250,427	35,76	2,401	2,109	Dairy starter	CP003157	8	188	[35]
*Lactococcus garvieae* ATCC49156*	1,950,135	38,80	2,024	1,947	Fish (*Alosa fallax*)	AP009332	-	0	[36]
*Lactococcus garvieae* Lg2*	1,963,964	38,80	2,045	1,968	Fish (*Alosa fallax*)	AP009333	-	0	[36]
Streptococcus thermophilus LMD-9**	1,856,368	39.08	1960	1743	Dairy starter	CP000419	2	132	[37]

* *Lactococcus garvieae* are fish pathogens

** *Streptococcus thermophilus* was used as a closely related outgroup in the analyses

Briefly, *Lactococcus garvieae* strains have the highest G+C content, ~38.80%, whereas the lowest G+C contents, ~34.86%, were from *L*. *lactis* NCDO 2118 and *L*. *lactis* KF147, both isolated from vegetables. Additionally, the genome sizes of the *Lactococcus* species range from ~1.95 Mb to ~2.60 Mb, and the two *L*. *garvieae* strains have the smallest genomes.

In this work, the only species harboring plasmids were *L*. *lactis* NCDO 2118, *L*. *lactis* KF147, *Lactococcus lactis* subsp. *lactis* KLDS 40325 and *Lactococcus lactis* subsp. *lactis* CV56 strains, *Lactococcus lactis* subsp. *cremoris* A76, *Lactococcus lactis* subsp. *cremoris* SK11 and *Lactococcus lactis* subsp. *cremoris* UC5099 (*L*. *cremoris* UC5099) strains, where the latter harbored the greatest number of plasmids (Table 1).

**Fig 1:**
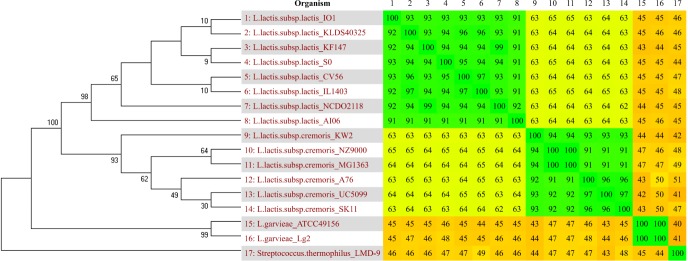
16S phylogenetic tree and genomic heatmap of *Lactococcus* genus. The *Streptococcus thermophilus* LMD-9 (position 17) was added to root the tree. The species in comparison are distributed from 1 to 17 in the same order, both vertically and horizontally. The numbers in the heatmap show the percentage of similarity between the species, varying from yellow (low similarity) to green (high similarity), or from 40% to 100%, respectively. The heatmap and the phylogenetic tree were created with the software Gegenees and Mega (Neighbor-Joining method with 1000 bootstraps replicates), respectively.

From the heatmap created with Gegenees (Fig 1), it is possible to visualize a high similarity between the subspecies of *Lactococcus*, with nucleotide similarities ranging from 40% to 100%. Additionally, the species and subspecies clustered separately, creating 3 green blocks of strains at the chart, represented by *L*. *lactis* subsp. *lactis* and *L*. *lactis* subsp. *cremoris*, with similarities ranging from 91% to 100%, and *L*. *garvieae*, in which the two strains of this species were 100% similar to each other.

On the phylogenetic tree created using 16S, the species and subspecies also clustered together, forming two main clades corresponding to the best similarity among *L*. *lactis* subsp. *lactis* and *L*. *lactis* subsp. c*remoris* (Fig 1). Additionally, *L*. *garvieae* strains appeared in an outside node compared to *L*. *lactis* species and are the two most distinct and distant species of *Lactococcus* on the heatmap and phylogenetic tree. Briefly, on the heatmap, the degree of intraspecies similarity varies from 91% to 100%, whereas interspecies similarity varies from 40% to 65%.

From the genome synteny analysis (S1 Fig), all strains from *L*. *lactis* subsp. *lactis* presented a high degree of synteny, where the most conserved genome compared to *L*. *lactis* NCDO 2118 (chosen as reference genome) was *L*. *lactis* KF147. Additionally, we performed a comparison with the plasmids of *L*. *lactis* NCDO 2118 and *L*. *lactis* KF147 strains. However, we verified a high degree of similarity from the beginning to the end of each plasmid sequence, meaning that they possibly harbored the same plasmid (data not shown).

### Metabolic pathways prediction

To identify conserved or non-conserved metabolic pathways, we used three different datasets, consisting of (1) the closely related *L*. *lactis* NCDO 2118, *L*. *lactis* KF147 and *L*. *lactis* IL1403, (2) all strains from *L*. *lactis* subsp. *lactis* and *L*. *lactis* subsp. c*remoris* (non-pathogenic dataset), and (3) all strains from this study (including *L*. *garviae*). The number of metabolic pathways harbored by each genome varies from 148 to 206, with a general mean of ~183 pathways. Both *L*. *garvieae* strains contained 148 metabolic pathways, *L*. *lactis* subsp. *lactis* showed an average of ~192 metabolic pathways, and *L*. *lactis* subsp. *cremoris* showed ~186 pathways.

The main differences were that the strain *L*. *lactis* NCDO 2118 contains more peptidoglycan biosynthesis pathways than *L*. *lactis* KF147 and *L*. *lactis* IL1403 strains. Other exclusive metabolic features of *L*. *lactis* NCDO 2118 in this context were complete anaerobic respiration pathways, fermentation of pyruvate to acetate, fermentation of fumarate, complete heterolactic fermentation, valine degradation, L-serine degradation, ammonia assimilation to glutamate, complete superpathway of acetate utilization and formation, protein degradation, initial pathway of sucrose degradation I, valine degradation, lysine degradation I and acyl-ACP thioesterase pathway (S1 Table).

### Genome plasticity

We identified 5 prophages in *L*. *lactis* NCDO 2118, of which 2 were incomplete, and 3 were considered intact (Table 2). The three intact phages harbored important genes such as *rusA*, *arsC1*, *arsC3*, *amtB*, *rpmE2*, *carA*, *pyrB*, *pyrP* and *pepT*.

**Table 2:** Intact and incomplete phages predicted in *L*. *lactis* subsp. *lactis* NCDO 2118.

Phages	Genes	Proteins
**Region 1 –*Intact phage***	*rusA e arsC1*	Integrase, Prophage, Phage antirepressor, Transcriptional regulator, Recombinase, Endodeoxyribonuclease, Aminotransferase, Phage terminase small subunit, Peptidase, Bacteriophage lysine, Arsenate reductase
**Region 2 –*Intact phage***	*amtB*, *kinA*, *llra*, *rpmE2*, *arsC3*, *carA*, *pyrB*, *pyrP*	Ammonium transporter, Sensor protein kinase, Two-component system regulator, 50S ribosomal protein L31 type B, Universal stress protein, Arsenate reductase, Bacteriophage lysine, Phage tail protein, Head-tail joining protein, Capsid protein, Phage ATP-dependent endopeptidase, Phage terminase small subunit, Endonuclease, Terminase, Replisome organizer, BRO-like protein, DNA binding protein, Phage integrase, Carbamoyl-phosphate synthase small chain, Aspartate carbamoyltransferase, Uracil transporter
**Region 3 –*Intact phage***	*pepT*, *ppaC*, *pflA*, *ysiA*, *ysiB*	Amino Acid permease, Peptidase T, Manganese-dependent inorganic pyrophosphatase, Pyruvate-formate lyase activating enzyme, Permease, Phage protein, Integrase
**Region 4 –*Incomplete phage***	*ardA*, *ecfA1*, *ecfA2*, *ecfT*, *dapH*, *yciA*	Peptidoglycan hydrolase, Antirestriction protein, Integrase, ATPase, Energy-coupling factor transporter, Thiol-disulfide isomerase, N-acetyldiaminopimelate deacetylase
**Region 5 –*Incomplete phage***	*glnA*	Integrase, Bacteriocin, DNA primase, Glutamine synthetase

Phage locations were predicted using the software PHAST.

**Fig 2:**
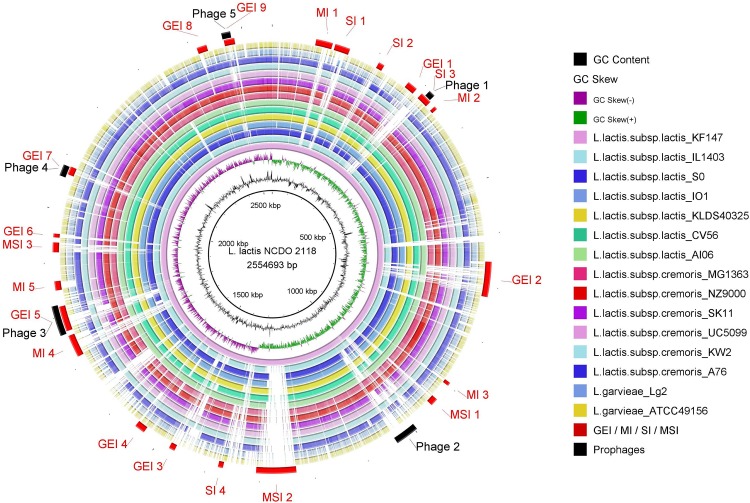
Circular comparison of the *Lactococcus* genus using *L*. *Lactis* NCDO 2118 as a reference. Each ring of the circle corresponds to a specific complete genome represented in the legend on the right. The similarity between species is represented by the intensity of the color. Darker colors represent higher similarities than bright ones. Deleted regions are represented by blank spaces inside the circles. (GEI = Genomic Island; MI = Metabolic Island; SI = Symbiotic Island; MSI = Miscellaneous Island, harboring both metabolic and symbiotic factors). Genomic islands and phage sequences were predicted with GIPSy and PHAST, respectively. The circular genomic comparisons were created with BRIG.

Additionally, we used BRIG to visualize the plasticity events from phage sequences (Fig 2). According to the BRIG analyses, phage 1 was incomplete in all species, except for the reference genome *L*. *lactis* NCDO 2118 and *L*. *lactis* KF147. Both phages 2 and 3, predicted as intact in the reference, were also present in *L*. *lactis* KF147, *L*. *lactis* IL1403, *Lactococcus lactis* subsp. *cremoris* NZ9000 and *L*. *cremoris* MG1363, whereas the former phage was also found in *Lactococcus lactis* subsp. *cremoris* KW2. Phage 4, also intact in the reference genome, was present in all other species. Phage 5, predicted as incomplete in the reference genome, was absent in *L*. *lactis* IO-1, *L*. *cremoris* KW2, *L*. *cremoris* UC5099 and partially present in both *L*. *garvieae* strains.

In the GIPSy predictions, we identified 9 Genomic Islands (GEIs), 5 Metabolic Islands (MIs), 4 Symbiotic Islands (SIs) and 3 Miscellaneous Islands (MSIs), which were predicted as harboring both metabolic and symbiotic related factors. The GEIs are listed in S2 Table.

All SIs were only partially present in the other strains, except for SI4, which was absent from all *L*. *garviae* strains, *L*. *lactis* subsp. *cremoris* strains and *L*. *lactis* IL 1403 (Fig 2). Additionally, all MIs presented regions of deletions in the pathogenic species *L*. *garviae*. The most prominent GEIs were MI3, which was only present in the two strains isolated from plants (*L*. *lactis* NCDO 2118 and *L*. *lactis* KF147), and MSI 2, which presented the biggest region of deletion in all *Lactococcus*, except for *L*. *lactis* NCDO 2118 and *L*. *lactis* KF147.

### Antibiotic resistance

LAB that are widely used as probiotics or in starter cultures have the potential to host antibiotic resistance genes, thereby presenting a risk of transferring such genes to many lactic acid bacteria and other pathogenic bacteria [23]. In the antibiogram assay, *L*. *lactis* NCDO 2118 was susceptible to ceftriaxone, erythromycin, tetracycline, ampicillin, penicillin and chloramphenicol and resistant to vancomycin, oxacillin and amikacin antibiotics (Table 3). Additionally, we tried to correlate the antibiogram profile with the genome content of *L*. *lactis* NCDO 2118, which presented 22 antibiotic resistance-related genes putatively coding for a VanZ family protein (NCDO2218_1094), penicillin-binding proteins (NCDO2118_0402, NCDO2118_0445, NCDO2118_0526, NCDO2118_0880 and NCDO2118_2216), and multidrug efflux pump proteins (Table 4). Additionally, no antibiotic resistance related gene presented deviation in its genomic signature.

**Table 3:** Antibiotic susceptibility of *L*. *lactis* NCDO 2118.

Antibiotic susceptibility assay			
Antibiotic	Concentration	Inhibition zone diameter (mm)	Susceptibility*
Ceftriaxone	30 μg	31	S
Erythromycin	10 μg	31	S
Tetracycline	30 μg	25	S
Ampicillin	30 μg	35	S
Vancomycin	10 U	0	R
Penicillin	30 μg	35	S
Amikacin	30 μg	15	R
Chloramphenicol	30 μg	28	S
Oxacillin	1 μg	14	R

* R = resistant, S = susceptible.

**Table 4:** Genes putatively coding for antibiotic resistance-related proteins.

Query ID	Product	Gene	G+C Content	Codon Usage
**NCDO2118_0089**	Multidrug resistance protein	*sugE*	NORMAL	NORMAL
**NCDO2118_0090**	Multidrug efflux transporter	*blt*	NORMAL	NORMAL
**NCDO2118_0108**	Multidrug resistance efflux pump	*pmrB*	NORMAL	NORMAL
**NCDO2118_0144**	MFS transporter	*ybfD*	NORMAL	NORMAL
**NCDO2118_0258**	Multidrug resistance ABC transporter	*-*	NORMAL	NORMAL
**NCDO2118_0259**	Multidrug ABC transporter ATP-binding protein	*-*	NORMAL	NORMAL
**NCDO2118_0363**	MFS transporter	*napC*	NORMAL	NORMAL
**NCDO2118_0369**	Multidrug ABC transporter ATP-binding protein	*lmrC*	NORMAL	NORMAL
**NCDO2118_0370**	Multidrug transporter	*lmrD*	NORMAL	NORMAL
**NCDO2118_0402**	Penicillin-binding protein 2B	*pbp2B*	NORMAL	NORMAL
**NCDO2118_0445**	Penicillin-binding protein 1B	*pbp1B*	NORMAL	NORMAL
**NCDO2118_0526**	Penicillin-binding protein 1A	*ponA*	NORMAL	NORMAL
**NCDO2118_0593**	Multidrug transporter	*-*	NORMAL	NORMAL
**NCDO2118_0645**	Multi-drug resistance efflux pump	*pmrA*	NORMAL	NORMAL
**NCDO2118_0726**	Multidrug resistance ABC transporter ATP-binding and permease protein	*lmrA*	NORMAL	NORMAL
**NCDO2118_0880**	Penicillin-binding protein 2X	*pbpX*	NORMAL	NORMAL
**NCDO2118_0930**	Multidrug resistance protein B	*-*	NORMAL	NORMAL
**NCDO2118_1094**	VanZ family protein	*-*	NORMAL	NORMAL
**NCDO2118_1401**	Multidrug MFS transporter	*-*	NORMAL	NORMAL
**NCDO2118_1736**	Multidrug transporter	*yqiA*	NORMAL	NORMAL
**NCDO2118_1995**	MFS transporter permease	*yteD*	NORMAL	NORMAL
**NCDO2118_2216**	Penicillin-binding protein 2a	*pbp2A*	NORMAL	NORMAL

G+C content and codon usage information were retrieved from GIPSy analyses.

### Identification of genes involved in acid stress and bile salt resistance

We searched the genome sequence of *L*. *lactis* NCDO 2118 for genes previously shown to be differentially expressed on cells cultivated under low and optimum pH (5.1 and 6.5, respectively) in *L*. *cremoris* MG1363 [38] (Table 5). Additionally, we also searched for genes differentially regulated by bile exposure in *Bifidobacterium animalis* and *Bifidobacterium longum* NCIMB 8809 [39; 40] and/or identified on the total proteome and surfome of *Lactobacillus rhamnosus* GG using proteomics analyses (Table 5). Here, we identified some genes in *L*. *lactis* NCDO 2118 that were previously reported to be involved in the acid stress response, including genes coding for chaperones (*dnaK*) and stringent response. Additionally, DnaK and Enolase are plasminogen receptors involved in bile modulation during intestinal colonization.

**Table 5:** Genes coding for proteins involved in acid stress and bile salt resistance.

Locus_tag	EC Number	Gene	Product	Stress response
NCDO2118_1870	-	*atpC*	ATP synthase epsilon chain	Acid stress
NCDO2118_1871	3.6.3.14	*atpD*	ATP synthase subunit beta	Acid stress
NCDO2118_1872	-	*atpG*	ATP synthase gamma chain	Acid stress
NCDO2118_1873	3.6.3.14	*atpA*	ATP synthase subunit alpha	Acid stress
NCDO2118_1874	-	*atpH*	ATP synthase subunit delta	Acid stress
NCDO2118_1875	-	*atpF*	ATP synthase subunit b	Acid stress
NCDO2118_1876	-	*atpB*	ATP synthase subunit a	Acid stress
NCDO2118_1877	-	*atpE*	ATP synthase subunit C	Acid stress
NCDO2118_1384	1.1.1.27	*ldh*	L-lactate dehydrogenase	Acid stress
NCDO2118_0475	-	*ptcC*	PTS system, cellobiose-specific IIC component	Acid stress
NCDO2118_0542	1.2.1.12	*gapA*	Glyceraldehyde-3-phosphate dehydrogenase	Acid stress
NCDO2118_0399	5.4.2.11	*gpmA*	2,3-bisphosphoglycerate-dependent phosphoglycerate mutase	Acid stress/bile resistance
NCDO2118_2272	5.3.1.9	*pgi*	Glucose-6-phosphate isomerase	Acid stress
NCDO2118_0096	2.7.1.40	*pyk1*	Pyruvate kinase	Acid stress
NCDO2118_1385	2.7.1.40	*pyk2*	Pyruvate kinase	Acid stress
NCDO2118_0240	2.7.2.3	*pgk*	Phosphoglycerate kinase	Acid stress/bile resistance
NCDO2118_0417	-	*recA1*	Protein RecA	Acid stress
NCDO2118_1251	-	*recA2*	Protein RecA	Acid stress
NCDO2118_0540	-	*clpE*	ATP-dependent Clp protease ATP-binding subunit	Acid stress
NCDO2118_0453	-	*groL*	60 kDa chaperonin	Acid stress
NCDO2118_1545	-	*clpB*	Chaperone protein	Acid stress
NCDO2118_0467	1.15.1.1	*sodA*	Superoxide dismutase	Acid stress
NCDO2118_0073	2.7.6.5	*relA*	GTP pyrophosphokinase	Acid stress
NCDO2118_0637	4.2.1.11	*eno*	Enolase	Acid stress/bile resistance
NCDO2118_1019	-	*dnaK*	Chaperone protein	Acid stress/bile resistance
NCDO2118_1594	3.5.99.6	*nagB*	Glucosamine-6-phosphate deaminase/isomerase	Bile resistance
NCDO2118_1909	3.4.24.-	*pepO*	Endopeptidase O	Bile resistance
NCDO2118_0941	5.4.99.9	*glf*	UDP-galactopyranose mutase	Bile resistance
NCDO2118_0500	6.3.4.2	*pyrG*	CTP synthase	Bile resistance
NCDO2118_0035	1.8.1.4	*pdhd*	Pyruvate dehydrogenase	Bile resistance
NCDO2118_2145	6.1.1.19	*argS*	Arginyl-tRNA synthetase	Bile resistance
NCDO2118_1958	-	*oppA*	Oligopeptide-binding protein	Bile resistance
NCDO2118_2203	-	*rpsC*	30S ribosomal protein S3	Bile resistance
NCDO2118_2191	-	*rpsE*	30S ribosomal protein S5	Bile resistance
NCDO2118_2208	-	*rplD*	50S ribosomal protein L4	Bile resistance
NCDO2118_2197	-	*rplE*	50S ribosomal protein L5	Bile resistance
NCDO2118_2193	-	*rplF*	50S ribosomal protein L6	Bile resistance

Additionally, we assayed *L*. *lactis* NCDO 2118 to see how it responds to the challenges of acid pH and bile salt secretion in the gastrointestinal tract. When in contact with artificial gastric juice, 48% of the *L*. *lactis* NCDO 2118 was not inhibited and was able to grow after acid pH challenge, whereas the contact with bile salts inhibited 95% of the bacteria growth, showing a high sensibility, as a result of three independent experiments (S2 Fig).

### Identification of genes coding for adhesins and adhesion-related proteins

Based on literature data, we predicted proteins involved in the adhesion mechanisms of *L*. *lactis* NCDO 2118, shown in Table 6. *L*. *lactis* NCDO 2118 harbors 19 genes putatively coding for adhesion-related proteins, such as the gene *chiA* (NCDO2118_2053) and the genes coding for the *Chitin binding protein* (CBP–NCDO2118_2054) and the laminin-binding protein (NCDO2118_1446).

**Table 6:** Proteins potentially involved in the adhesion mechanisms of *L*. *lactis*.

Locus_tag	Gene	Product
NCDO2118_0315		Hypothetical protein
NCDO2118_0552		Hypothetical protein
NCDO2118_0647	*pycA*	Pyruvate carboxylase
NCDO2118_0684		ChW repeat-/cell adhesion domain-containing transglutaminase-like protease
NCDO2118_0727		Hypothetical protein
NCDO2118_0774		Flagellar hook-length control protein FliK
NCDO2118_0776		Hypothetical protein
NCDO2118_0806	*exoA*	Exodeoxyribonuclease
NCDO2118_0857		Hypothetical protein
NCDO2118_1205		Hypothetical protein
NCDO2118_1365		Hypothetical protein
NCDO2118_1446	*bmpA*	Basic membrane protein A (laminin-binding protein)
NCDO2118_1515	*ypdD*	Sugar hydrolase
NCDO2118_1627		Hypothetical protein
NCDO2118_2053	*chiA*	Chitinase
NCDO2118_2054		Chitin binding protein
NCDO2118_2211		Hypothetical protein
NCDO2118_2278		Fibronectin-binding protein
NCDO2118_2284		Hypothetical protein

To determine whether *L*. *lactis* NCDO 2118 exhibited adhesive ability, corroborating the *in silico* data, we performed microbial adhesion to solvents (MATS) experiments, which demonstrated a moderate cell surface hydrophobicity, as suggested by Nader-Macías *et al*., (2008) [41], with 52% association with xylene.

### Bacteriocins and other competitive exclusion mechanisms

To predict putative bacteriocins, we used the software BAGEL [42]. In addition to identification, BAGEL also classifies the bacteriocins into three classes: (i) lanthionine-containing bacteriocins/lantibiotics, (ii) non-lanthionine-containing bacteriocins and (iii) bacteriolysins/non-bacteriocin lytic proteins [43].

**Fig 3:**
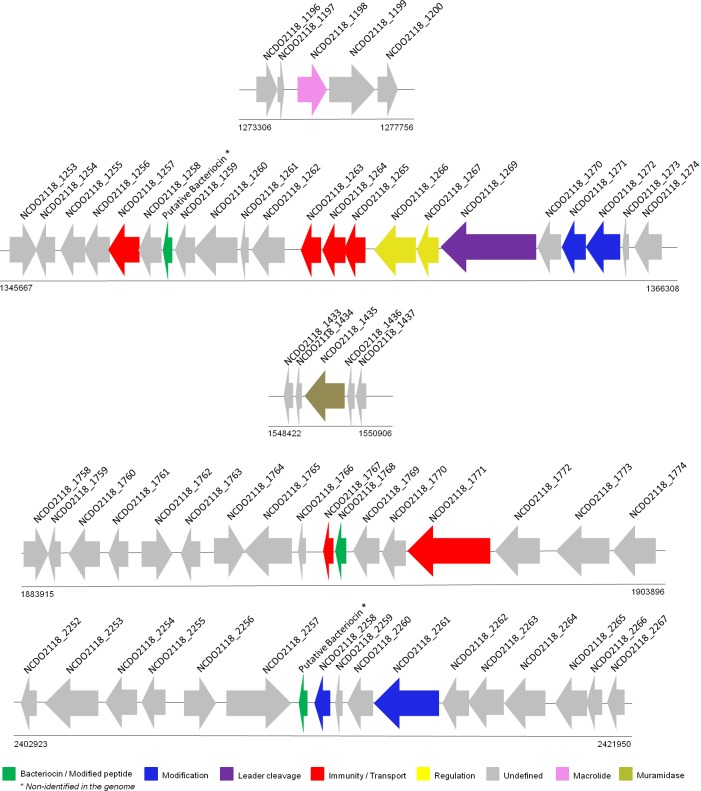
Regions of bacteriocins predicted with BAGEL in *L*. *lactis* NCDO 2118. BAGEL predicted three putative bacteriocins, one of each class. (A) Putative bacteriocin/Class I predicted on orf010 (pseudogene) and *nisZ* was found with manual curation. (B) Putative bacteriocin/Class II predicted on orf027 (pseudogene). (C) Putative bacteriocin/Sactipeptidase predicted on orf011 (this region was not previously characterized in the *L*. *lactis* subsp. *lactis* NCDO 2118 genome). All putative bacteriocins were also identified in Bactibase.

In *L*. *lactis* NCDO 2118, BAGEL predicted one bacteriocin for each of the three classes (Fig 3): a lanthipeptide (class I), NCDO2118_1768 (putative Bacteriocin-lactococcin-A—class II) and a putative bacteriocin (class III), located between NCDO2118_2257 and NCDO2118_2258. The class III putative bacteriocin was not described in the *L*. *lactis* NCDO 2118 genome, possibly because the gene-finding methodology failed to predict it. The bacteriocin of class I is a lantibiotic Nisin coded by the *nisZ* gene (NCDO2118_1272), a natural variant of *nisA* [44]. Briefly, Nisin is commonly produced by strains of *L*. *lactis*, and the cluster of genes coding for the nisin biosynthesis proteins consists of 11 genes: *nisABTCIP* (biosynthesis and immunity), nis*FEG* (immunity) and the two-component regulatory system *nisRK* [45]. *L*. *lactis* NCDO 2118 harbors a *nisBCIP* operon (where *nisP* is a pseudogene), a *nisRK* two-component system and a *nisFEG* operon. Additionally, BAGEL has predicted the presence of another putative bacteriocin between NCDO2118_1258 and NCDO2118_1259 that is located close to the class I cluster of genes. However, the amino acid sequence predicted from this region only presents similarity to a hypothetical protein. Lactococcin A is a class IId, non-pediocin-like, single-peptide bacteriocin normally produced by strains of *L*. *lactis*. Four genes are responsible for the biosynthesis of lactococcin: the lactococcin-A coding gene, one immunity gene and the dedicated ABC transporter system along with its accessory protein. *L*. *lactis* NCDO 2118 harbors an immunity protein (NCDO2118_1767) and lactococcin-A (NCDO2118_1768). As for the class III prediction, the predicted putative bacteriocin is located upstream of two hypothetical proteins (NCDO2118_2258 and NCDO2118_2259); however, little is known about the organization of the gene cluster of class III bacteriocins [45], and the putative bacteriocin predicted by BAGEL only presents similarity to hypothetical proteins in GENBANK.

Moreover, an additional bacteriocin-coding gene was harbored by GEI 9 (S2 Table), which was not predicted by BAGEL. Through blast analyses, we found a significant amino acid similarity, with identities varying from 76% to 98%, between this gene and a bacteriocin-coding gene from other *L*. *lactis* in the UNIPROT and NCBI BLAST databases. However, many of the genes were also described as hypothetical proteins. In addition, we also searched for other genes that could possibly play a role in the competitive exclusion of other bacteria. A *Lyzozyme M1* and a Macrolide biosynthetic protein encoding genes were also included in S3 Table after manual curation in the *L*. *lactis* NCDO 2118 genome.

In the present study, a deferred agar spot assay was used for the initial determination of antagonistic activity via diffusible compound(s) produced by *L*. *lactis* NCDO 2118. To assay whether *L*. *lactis* NCDO 2118 could affect the growth of pathogenic bacteria, we used an approach to measure its antagonistic activity against the strains *Salmonella enterica* ATCC 14028, *Escherichia coli* ATCC 25723, *Staphylococcus aureus* 29213, *Bacillus cereus* ATCC 11778, *Listeria monocytogenes* ATCC 15313, *Enterococcus faecalis* ATCC 19433 and *Pseudomonas aeruginosa* ATCC 5853. *L*. *lactis* NCDO 2118 showed no effect on the growth of the abovementioned pathogenic strains.

### In silico identification of putatively secreted proteins

Here, we strove to predict genes encoding secreted proteins from *L*. *lactis* NCDO 2118 that are absent from the genomes of the strains *L*. *lactis* IL1403 and *L*. *cremoris* MG1363, as the secreted proteins of *L*. *lactis* NCDO 2118 are possibly responsible for the immunomodulatory effects of this transient bacterium inside the host [18].

**Fig 4:**
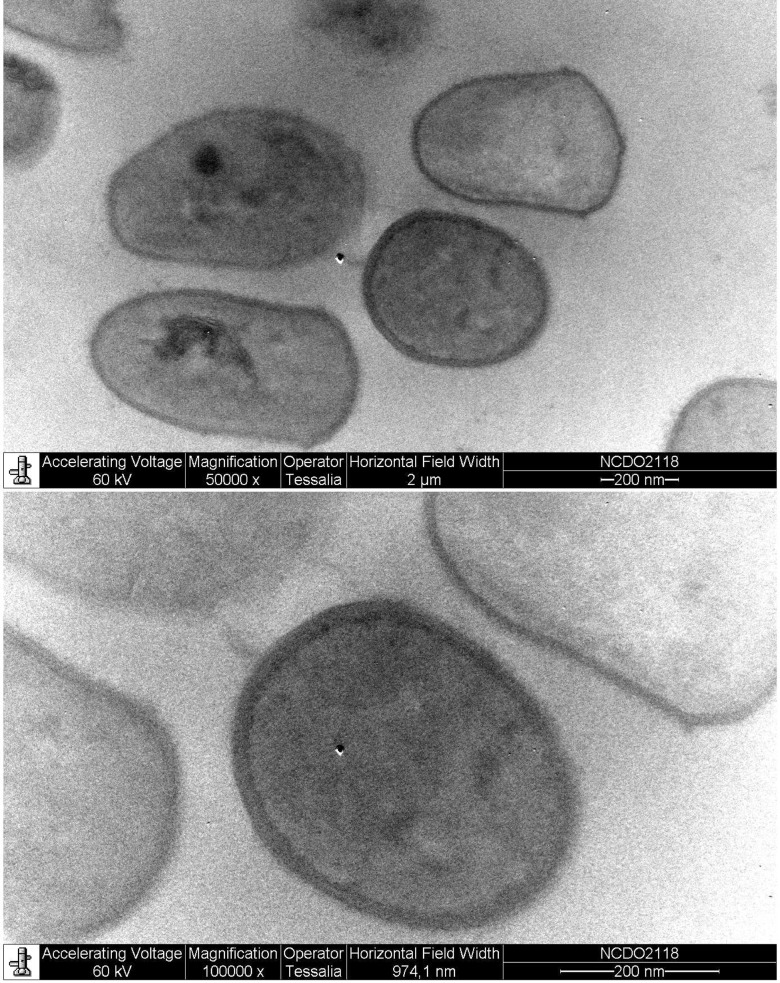
Photomicrograph of *L*. *lactis* NCDO 2118. The measurements of the membrane wall were performed with ImageJ software using images generated with electron microscopy with EM10A equipment (Zeiss). Top: magnification of 50,000 times; bottom: magnification of 100,000 times.

To predict the secreted proteins, we used the software SurfG+, which classifies the proteins using an identification approach based on the presence/absence of signal peptides, signal retention and transmembrane helix [46], which are correlated with the cell wall thickness of the bacteria. To determine the cell wall thickness, we made photomicrographs of *L*. *lactis* NCDO 2118 (Fig 4); the cell wall was measured more than 270 times, showing an average size of ~20 nm, and this value was used to determine the motifs. If none of the motifs were found in the protein sequence, SurfG+ characterized the protein as cytoplasmic (CYT) [47]. Using SurfG+, we predicted 94 secreted proteins in *L*. *lactis* NCDO 2118.

From this data, the secreted proteins of *L*. *lactis* NCDO 2118 were compared to the proteins identified in *L*. *lactis* IL1403 using OrthoMCL [48]. In this comparison, 26 of the secreted proteins were exclusive from *L*. *lactis* NCDO 2118. Because the probiotic effect was searched using secreted proteins previously expressed *in vitro*, we searched for proteins that were expressed in *L*. *lactis* NCDO 2118 *in vitro* using proteomics analyses. Five proteins were both present in the 26 secreted proteins that were exclusive from *L*. *lactis* NCDO 2118 and in the 867 expressed proteins from proteomic analyses (Table 7). The complete lists of genes identified in proteomic analyses, in the prediction of subcellular location and the exclusive proteins of *L*. *lactis* NCDO 2118 are described in S4 Table.

**Table 7:** Prediction of exclusive secreted proteins of *L*. *lactis* NCDO 2118.

Locus tag	Gene	Start	Stop	Product	Orthology/Subcellular Location/Proteome
NCDO2118_0052	NCDO2118_0052	57803	58270	Hypothetical protein	Exclusive/ Secreted
NCDO2118_0128	*epsX*	133945	134712	Polysaccharide biosynthesis protein	Exclusive/ Secreted
NCDO2118_0139	*epsK*	144750	145652	Polysaccharide biosynthesis protein	Exclusive/ Secreted/ Expressed
NCDO2118_0140	*epsL*	145677	146600	Transcriptional regulator	Exclusive/ Secreted/ Expressed
NCDO2118_0212	NCDO2118_0212	214606	215988	Hypothetical protein	Exclusive/ Secreted/ Expressed
NCDO2118_0256	NCDO2118_0256	255719	256297	Hypothetical protein	Exclusive/ Secreted
NCDO2118_0291	NCDO2118_0291	285998	287113	Endoglucanase	Exclusive/ Secreted
NCDO2118_0294	NCDO2118_0294	288612	289397	Hypothetical protein	Exclusive/ Secreted
NCDO2118_0483	NCDO2118_0483	478392	479351	Hypothetical protein	Exclusive/ Secreted
NCDO2118_0533	NCDO2118_0533	527774	527965	Hypothetical protein	Exclusive/ Secreted
NCDO2118_0683	NCDO2118_0683	697697	698158	Hypothetical protein	Exclusive/ Secreted/ Expressed
NCDO2118_0684	NCDO2118_0684	698177	701176	ChW repeat-/cell adhesion domain-containing transglutaminase-like protease	Exclusive/ Secreted
NCDO2118_0882	NCDO2118_0882	918428	918706	Hypothetical protein	Exclusive/ Secreted
NCDO2118_0904	NCDO2118_0904	939391	940704	Hypothetical protein	Exclusive/ Secreted
NCDO2118_0942	NCDO2118_0942	985414	986700	Hypothetical protein	Exclusive/ Secreted
NCDO2118_0991	NCDO2118_0991	1034860	1035321	Hypothetical protein	Exclusive/ Secreted
NCDO2118_1361	NCDO2118_1361	1468537	1469364	Hypothetical protein	Exclusive/ Secreted
NCDO2118_1363	NCDO2118_1363	1474372	1475115	Hypothetical protein	Exclusive/ Secreted
NCDO2118_1364	NCDO2118_1364	1475137	1475901	Hypothetical protein	Exclusive/ Secreted
NCDO2118_1420	NCDO2118_1420	1537567	1538400	Hypothetical protein	Exclusive/ Secreted/ Expressed
NCDO2118_1459	NCDO2118_1459	1569307	1569477	Hypothetical protein	Exclusive/ Secreted
NCDO2118_1795	NCDO2118_1795	1927992	1929140	Transcriptional regulator	Exclusive/ Secreted
NCDO2118_2077	NCDO2118_2077	2227730	2228593	Hypothetical protein	Exclusive/ Secreted
NCDO2118_2151	NCDO2118_2151	2304776	2305051	Hypothetical protein	Exclusive/ Secreted
NCDO2118_2232	NCDO2118_2232	2371307	2372062	Hypothetical protein	Exclusive/ Secreted
NCDO2118_2330	NCDO2118_2330	2482143	2482712	Hypothetical protein	Exclusive/ Secreted

Exclusive, secreted and expressed proteins were predicted using OrthoMCL, SurfG+ and proteomic analyses, respectively.

## Discussion

### Genomic characterization of *L*. *lactis* NCDO 2118 and comparison with other species

The genomic lengths of the *Lactococcus* species analyzed here are highly variable (from ~1.95 Mb to ~2.60 Mb). However, the finding that *L*. *garvieae* strains have the smallest genomes compared to *L*. *lactis* strains is in agreement with the lifestyle of *L*. *garvieae*, isolated from diseased fish. Because pathogenic bacteria may scavenge compounds from the host for their own metabolism, they tend to lose genes involved in biosynthetic pathways, thus, presenting smaller genomes [49].

The high similarity at the subspecies level may be related with some specific characteristics already described in literature, such as the propensity of *L*. *lactis* subsp *lactis* to form longer chains. Besides, *L*. *lactis* subsp. *lactis* are able to produce GABA, ammonia from arginine, carbon dioxide and diacetyl formation from citrate as opposing to *L*. *lactis* subsp. *cremoris* subspecies. Additionally, analyses using southern hybridization, PFGE, 16 rRNA and housekeeping genes (*atpA*, *rpoA*, *pheS*, *pepN*, *bcaT*, *pepX*) showed two separate clusters formed by *L*. *lactis* subsp. *lactis* and *L*. *lactis* subsp. *cremoris* with a low degree of similarity between them [50–52].

From the genome synteny analyses, we have found a high degree of synteny between *L*. *lactis* KF147 and *L*. *lactis* IL1403, which was already reported in a previous work [25]. However, there was no other genome sequence of any *Lactococcus* species correlated with plants available at the time the work was performed. Here, we found that the most conserved genome compared to *L*. *lactis* NCDO 2118 was *L*. *lactis* KF147. The material of fermented plant covers a highly variable niche according to some characteristics as: chemical composition and physical conditions. Thus, plant-related strains posses a great metabolic diversity that certainly extrapolates that from dairy strains [53].

Finally, although *L*. *lactis* NCDO 2118 shares several pathways in common with *L*. *lactis* KF147 and *L*. *lactis* IL1403, it presents several exclusive metabolic features that may be explored for future utilization in industry.

### Evaluation of safety aspects in the use of *L*. *lactis* NCDO 2118 by genome plasticity and antibiotic resistance approaches

Plasmid-linked antibiotic resistance is not very common among LAB, but it does occur, and safety implications should be taken into consideration. Strains harboring resistance plasmids should not be used as human or animal probiotics. Checking the ability of a proposed probiotic strain to act as a donor for conjugative antibiotic resistance genes may be a sensible precaution in some instances [54].

To provide a better understanding of the putative plasticity of *L*. *lactis* NCDO 2118, we have predicted putative phage and genomic islands of this species. The presence of phage regions may contribute to the acquirement of antibiotic resistance, the ability to survive in a new environment, the improvement of adhesion ability, or even to turning the bacteria pathogenic [55]. Here, we found 5 phages; the 3 intact phages harbored important genes such as *rusA*, *arsC1*, *arsC3*, *amtB*, *rpmE2*, *carA*, *pyrB*, *pyrP* and *pepT*. The *rusA* gene is associated with the prophage sequences of several genera of bacteria, including *Bacillus*, *Streptococcus*, *Staphylococcus*, and *Enterococcus*, and it is also present in *Lactococcus lactis* phage r1t [56]. The *arsC1* gene is related to arsenate resistance in *Corynebacterium glutamicum* [57]. *arsC3* codes for a thioredoxin-dependent arsenate reductase of the *Mycobacterium* sp. A33 [58]. *amtB* is a gene of the ammonia transporter family, which is found in eubacteria, archaea, fungus, plants and animals, whereas in prokaryotes, its homologue is co-transcribed with a PII paralogue, GlnK, in response to nitrogen limitation [59]. The *rpmE2* gene codes for a L31 ribosomal protein. The genes *carA*, *pyrB* and *pyrP* are organized as an operon in *L*. *cremoris* MG1363, where *pyrP* encodes a membrane-bound protein with high affinity to uracil permease and pyrimidines, and *pyrB* and *carA* encode pyrimidine biosynthetic enzymes [60]. Finally, the gene *pepT* encodes for a tripeptidase.

Additionally, we predicted 9 GEIs, 5 MIs, 4 SIs and 3 MSIs in the genome sequence of *L*. *lactis* NCDO 2118. Interestingly, all MIs present deletions in the pathogenic species *L*. *garvieae*, which is a common feature of pathogenic bacteria that adapted to scavenge nutrients from the host [61]. Additionally, MI3 is only present in the *L*. *lactis* NCDO 2118 and *L*. *lactis* KF147 and may be important for the adaptation of those strains to plants.

We have also assayed *L*. *lactis* NCDO 2118, aiming to characterize its antibiotic resistance profile. *L*. *lactis* NCDO 2118 is susceptible to most of the antibiotics assayed here. Although *L*. *lactis* NCDO 2118 presented resistance to oxacillin and susceptibility to penicillin, it only harbored genes coding for a VanZ family protein, which may be related to the vancomycin resistance, penicillin-binding proteins, and multidrug efflux pump proteins.

The efflux pumps are membrane transporter proteins responsible for the extrusion of relevant antibiotics, which are found in both Gram-positive and Gram-negative bacteria [62; 63]. Penicillin-binding proteins are transpeptidases or caboxypeptidases that harbor specific motifs that limit the active site serine penicillin-recognizing enzyme family, including class A and C β-lactamases [64]. Vancomycin is a glycopeptide antibiotic used in severe infections. Some species used in the food industry or found naturally in raw food material present an intrinsic resistance to vancomycin, including *L*. *rhamnosus*, *L*. *casei*, *Lactobacillus plantarum*, and *Leuconostoc lactis* [65].

Finally, although *L*. *lactis* NCDO 2118 does present genes putatively coding for antibiotic resistance-related proteins, none of those genes present anomalous G+C or codon usage deviation, nor are they harbored by the putative horizontally acquired regions predicted by GIPSy or PHAST. More interestingly, no Resistance Island was identified in *L*. *lactis* NCDO 2118, corroborating its safety aspects [66].

### In vitro and in silico analyses of survival, exclusion mechanisms and probiotic properties of *L*. *lactis* NCDO 2118

#### Susceptibility of *L*. *lactis* NCDO 2118 to acid stress and bile salts

Concerning the acid stress, lowering the intracellular pH reduces the transmembrane pH difference and the activity of acid-sensitive enzymes and damages proteins and DNA [67].The first mechanism used by *L*. *lactis* species to cope with acid stress is to maintain a low intracellular pH (pHi) by using membrane ATPase FoF1 [68; 69] and the generation of alkaline substances through the catabolism of amino acids (deamination, for example) [70; 71]. Bile salts, on the other hand, are surface-active, amphipathic molecules with a potent antimicrobial activity, and they act as detergents that disrupt biological membranes [67]. The percentage of resistance to bile salts also tends to vary among LAB and even between strains of the same species [72].

Here, we have identified 25 and 16 genes previously shown to be involved in acid stress and bile resistance in other species, respectively. In an *in vitro* assay, however, only 48% of *L*. *lactis* NCDO 2118 was able to grow after pH challenge, and 95% of bacteria was inhibited by bile salts. Other authors have already found that bacteria with an intestinal origin tend to be more resistant to stomach acids [73]. Therefore, this finding corroborates our results because *L*. *lactis* NCDO 2118 was isolated from frozen peas. Most of the genes found in *L*. *cremoris* MG1363 were also identified in *L*. *lactis* NCDO 2118. Additionally, a work using proteomics analyses identified some genes related to acid response and they are present in *L*. *lactis* NCDO 2118 genome (*clpEP*, *ahpC*, *tig*, *hpr and luxS*) [74] showing that other approaches may better elucidate the mechanism of survival to acid stress on this strain.

The high susceptibility of *L*. *lactis* NCDO 2118 to bile salts, on the other hand, must be further explored *in vitro* and *in vivo* using transcriptomics analyses to determine the expression rates of the described genes.

#### Competitive exclusion mechanisms of *L*. *lactis* NCDO 2118

There are several mechanisms used by bacteria to competitively exclude other species, such as bacteriocin production, space competition through the use of adhesins or receptors that bind to specific surface features, predation and even rapid growth [75].

Adhesins are responsible for the recognition and colonization of host tissues through specific binding. This process may activate the innate host cells or the expression of new genes. Adhesins may be characterized as hair-like attachments named pili or fimbriae or in other cases, named non-pilus adhesin, related to the microbial cell surface [76].

In *L*. *lactis* NCDO 2118, we have identified the gene *chiA* (NCDO2118_2053) and the genes coding for the *Chitin binding protein* (CBP–NCDO2118_2054) and the laminin-binding protein (NCDO2118_1446), which are normally related to adhesion in other bacteria. Chitin is degraded by chitinases that belong to members of the glycoside hydrolase of family 18 [77]. One example of bacteria that produces chitinase is *Serratia marcescens*, one of the most efficient organisms in chitin degradation [78]. When *E*. *coli* was cloned with a chitin-binding protein of *Serratia marcescens*, there was a significant increase in its ability to adhere to human colon cells [77].

Chitin-binding encoding genes are broadly distributed in many microorganisms. The *L*. *lactis* IL1403 genome, for example, harbors chitinolytic machinery represented by one family 33 CBP (*yucG;* referred as *Ll*CBP33A), one family 18 chitinase (*chiA*, referred as *Ll*Chi18A) and one family 20 *N-*acetylhexosaminidase [3; 79]. Another example of bacteria that present a high adhesion degree is *Borrelia burgdorferi*, which is able to bind to mammalian laminin, an important extracellular matrix (ECM) component [80]. A laminin-binding protein has also been identified in *L*. *lactis* NCDO 2118.

Additionally, we have found using MATS experiments that *L*. *lactis* NCDO 2118 presents a 52% of association to xylene, which supports the presence of genes coding for adhesion-related proteins in this strain. The hydrophobicity is directly related to the capacity of strains to adhere to surfaces. This capacity is determined by hydrophobic components present in the outer membrane of microorganisms, and it is known that hydrophobic interactions have an important role in the adhesion of bacteria to the epithelium. The application of MATS experiments facilitates a qualitative assessment of the polarity or non-polarity of the bacterial surface, which is important because it indicates the potential for probiotic adhesion to apolar surfaces in the intestinal and vaginal epithelia. However, this test is only a primary indicator of the adherence of microorganisms [81; 82].

The other bacterial competitive exclusion mechanism assayed here was the production of exclusion antimicrobial peptides, named bacteriocins. Bacteriocins produced by a bacterium may be activated against others, even ones from the same species, while the producer is immune to its own peptides [43]. This exclusion mechanism is very important for probioses, as it renders probiotic organisms able to compete with and kill pathogenic ones, promoting a health benefit to the host [2]. We have predicted one bacteriocin for each of the three classes in *L*. *lactis* NCDO 2118 (class I-III), which may be important for exclusion mechanisms of this bacteria. However, the lack of *nisT* and the pseudogenization of *nisP* on the class I gene cluster, the lack of ABC-transporters in the class II cluster and, also, the lack of information regarding the product of the putative bacteriocin in the class III cluster have to be further studied using *in vitro* analyses to elucidate whether those bacteriocins are produced and present antimicrobial activity or not.

We have also performed a deferred agar spot assay for the initial determination of antagonistic activity produced by *L*. *lactis* NCDO 2118. This test indicates the activity against various Gram-positive and -negative bacteria. This inhibitory effect may be due to H_2_O_2_, lactic acid, bacteriocins, antibiotic-like substances, or a combination of these compounds [83]. However, *L*. *lactis* NCDO 2118 showed no effect on the growth of the pathogenic strains assayed here.

#### Secreted proteins and immunomodulatory effects

According to Luerce *et al*., (2014), the secreted proteins of *L*. *lactis* NCDO 2118 are possibly responsible for the immunomodulatory effects of this transient bacterium inside the host. In a comparison of the anti-inflammatory effects between *L*. *lactis* NCDO 2118, *L*. *lactis* IL1403 and *L*. c*remoris* MG1363 strains, only the *L*. *lactis* NCDO 2118 supernatant was able to decrease the IL-8 production (45%), showing its immunomodulatory ability against inflammation [18].

Here, we predicted 5 proteins that are present in the 26 secreted proteins exclusive from *L*. *lactis* NCDO 2118 and in the 867 expressed proteins from proteomic analyses and may thus be related to the probiotic effect of this strain (Table 7). From those 5 exclusive, secreted and expressed genes of *L*. *lactis* NCDO 2118, *epsK* and *epsL* are part of the operon *epsABCDEFGHIJKLX*, whereas there is an *epsR* gene located in another genomic region.

The EPSs are a type of biopolymer able to facilitate intense interactions of biofilm cells through adhesion, aggregation of bacterial cells, cohesion of biofilms, protective barriers, and cell component export [84]. Through microarray and electron microscopy analyses, Denou *et al*., 2008 found an *eps* cluster of genes exclusive from a probiotic *Lactobacillus* strain compared to a type strain and they have shown that deletion of this cluster from the probiotic strain results in lack of the fuzzy layer on the outside of the cell wall [85].

Altogether, the lack of further knowledge of the *eps* cluster of genes and the presence of three other genes coding hypothetical exclusive/secreted/expressed proteins highlight the need for additional studies to better elucidate the underlying mechanisms involved in the anti-inflammatory and immunomodulatory activities of this strain.

## Materials and methods

### Genome sequences

The genome sequences of *L*. *lactis* NCDO 2118 [24] and 15 other strains of *Lactococcus* were retrieved from the GENBANK dataset of NCBI (Table 1). Briefly, the dataset is composed of 8 strains of *Lactococcus lactis* subsp. *lactis*, 2 of which were isolated from legumes (*L*. *lactis* NCDO 2118 and *Lactococcus lactis* subsp. *lactis* KF147), 6 *Lactococcus lactis* subsp. *cremoris* isolated from dairy or other fermented foods, and 2 *Lactococcus garvieae* isolated from diseased fish. *L*. *garvieae* was added to the analyses because it is a closely related pathogenic species. *S*. *thermophilus* LMD-9 was used as an outgroup to root the phylogenetic tree. Only complete genomes were used to avoid bias.

### In silico analyses

#### Heatmap of genome similarities and 16S phylogenetic tree

The heatmap analyses of the 17 strains were performed with Gegenees [86]. The input files consisted of complete genomes in.fna format. *Streptococcus thermophilus* LMD-9, a closely related species, was used as an outgroup to root the tree. The analyses were performed with default parameters for comparative analyses using the alignment method BLASTn. Gegenees performs an all-versus-all alignment process of the fragments generated from the 17 genomes. The result was exported from Gegenees as a heatplot image. Additionally, a phylogenetic tree was made using the 16S sequences from all genomes as identified by RNAmmer [87]. After that, they were aligned in MUSCLE [88], and the phylogenetic tree was inferred using the Neighbor-Joining method with 1000 bootstrap replicates.

#### Genome synteny

The genome synteny analyses were performed using Mauve, with the "progressiveMauve" option and all genome sequences in the.fna format. Mauve predicts gene synteny by merging locally collinear blocks of conserved genome orthologous regions and ordering them according to a reference genome [89].

#### Genome plasticity

The genome plasticity analyses were performed by searching for horizontally acquired regions such as genomic islands and phage sequences. The genomic islands were searched using the software GIPSy: Genomic Island Prediction Software [90], which updates the methodology of the software PIPS: Pathogenicity Island Prediction Software. Briefly, GIPSy performs the prediction of four different classes of genomic islands: Pathogenicity Islands, Resistance Islands, Metabolic Islands and Symbiotic Islands. In this work, we searched for metabolic and symbiotic islands in the genome of *L*. *lactis* NCDO 2118 using *Lactococcus lactis* subsp. *cremoris* MG1363 and *Lactococcus garviae* Lg2 genomes as subjects. After, we consolidated and manually curated the results. The choice of metabolic and symbiotic islands was made based on the lifestyle of *L*. *lactis* NCDO 2118, a strain isolated from vegetables, and its metabolic importance.

All the analyses were performed using GENBANK files and default parameters. The results were exported in tabulated format and used in BRIG (Blast Ring Image Generator) to generate circular genome comparative views [91]. Finally, the prophage prediction was performed using the GENBANK file and the software Phast [92], and the results were exported in table format and used as input in BRIG.

#### Bacteriocin prediction

The bacteriocin prediction was performed in BAGEL software using the.fna file from *L*. *lactis* NCDO 2118. Briefly, the software works with a curated dataset of bacteriocins, in which the input data are evaluated based on a Hidden Markov Model. The genetic information is analyzed based on combinations of PFAM domains [42]. For the putative bacteriocin predicted on *L*. *lactis* NCDO 2118 (NCDO2118_1768), we used the Transporter Classification Database (TCDB) [93] with an e-value of e-07.

#### Circular comparison map of genomic sequences

To create circular genome comparisons, we used the software BRIG and all genome sequences in the.fna format; we created the figure with *L*. *lactis* NCDO 2118 as reference strain. Additionally, we added the coordinates of the genomic islands and phage regions to the figure to visualize genome plasticity events. Finally, all genomes underwent BLAST analyses against the reference strain to create the circular comparison map.

#### Metabolic pathway prediction

A genome sequence in.fasta and a genome annotation in the.gbk format were used for reconstructing the *Lactococcus* species metabolic pathways. Posteriorly, the Pathway/Genome Databases (PGDB) for each of the 16 strains were computationally predicted using Pathway Tools software version 16.5 [94], developed by SRI International. The MetaCyc, a highly curated and non-redundant reference database of small-molecule metabolism, was used as a reference database for the PathoLogic component of the Pathway Tools software [95]. The metabolic pathways of *L*. *lactis* NCDO 2118 were used as a reference for the comparative analysis using the following comparisons: i) *L*. *lactis* NCDO 2118, *L*. *lactis* KF147 and *Lactococcus lactis* subsp. *lactis* IL1403, ii) non-pathogenic strains of *L*. *lactis* (*L*. *lactis* subsp. *lactis* and *cremoris*), and iii) all strains in this study.

#### Identification of the secretome

The prediction of the putative subcellular localizations of *L*. *lactis* NCDO 2118 proteins was performed *in silico* using SurfG+. This software contains such tools as *SignalP*, *LipoP* and *TMHMM* for the identification of motifs [46]. Interestingly, SurfG+ uses the size of the membrane wall to better differentiate the membrane (MEM) and potentially surface exposed (PSE) proteins. Here, the measurements of the membrane wall were performed with electron microscopy with EM10A equipment (Zeiss), as previously described [96].

*L*. *lactis* NCDO 2118 was grown at 30°C for 18 h in M17 medium (Difco) containing 0.5% glucose [18] and then centrifuged. The resulting precipitate (~500 mL) was placed in an Eppendorf tube, fixed in 2.5% glutaraldehyde in 0.1 M sodium cacodylate buffer (pH 7.2) for 6 h at 8°C and washed three times with 0.1 M sodium cacodylate buffer (pH 7.2). After washing, the sample was post-fixed in 1% osmium tetroxide in 0.1 M sodium cacodylate buffer (pH 7.2) + 1.5% potassium ferrocyanide for 90 minutes, washed with 0.1 M with sodium cacodylate buffer (pH 7.2), dehydrated in a graduated ethanol series (50% EtOH, 70% EtOH, 95% EtOH, and 100% EtOH), and incorporated in Eponate–Araldite resin. Ultrathin sections were obtained using uranyl acetate and lead citrate and then verified by Zeiss-EM-10A [97]. The micrograph was obtained by one CCD Mega View camera. The thickness of the *L*. *lactis* NCDO 2118 wall was determined from the image analysis micrograph in ImageJ software (available at imagej.nih.gov/ij/).

To measure the wall, we used at least five micrographs of *L*. *lactis* NCDO 2118 with magnifications of 50,000 and 100,000 times. We calculated the mean size of the cell walls, and the average number of amino acids for the obtained wall thickness was ~55 amino acids. This value was added to the SurfG+ software together with the.fasta sequence of amino acids (.faa) exported from the strain of interest.

After this process, we used OrthoMCL tool to predict the orthologous and paralogous genes between *L*. *lactis* NCDO 2118 and *L*. *lactis* IL1403.

### In vitro analyses

#### Bacterial strains and growth conditions

For in vitro analyses, we used the probiotic strain *L*. *lactis* NCDO 2118 [18] and the pathogenic strains *Salmonella enterica* serovar *Typhimurium* ATCC 14028, *Escherichia coli* ATCC 25723, *Staphylococcus aureus* ATCC 29213, *Bacillus cereus* ATCC 11778, *Listeria monocytogenes* ATCC 15313, *Enterococcus faecalis* ATCC 19433, and *Pseudomonas aeruginosa* ATCC 25853.

*L*. *lactis* NCDO 2118 was grown at 37°C in MRS medium (Difco) without agitation for 18 hours. *L*. *monocytogenes* was cultured in TSB-YE for 24 hours at 28–30°C. The pathogenic strains were grown in BHI medium (BD) for 24 hours at 37°C. To prepare the solid and semi-solid culture media, we added 1.5% and 0.2–0.75% of agar, respectively.

#### *L*. *lactis* gastric juice susceptibility

*L*. *lactis* NCDO 2118 stationary phase cells were suspended in either 0.9% saline solution (pH 7) or simulated gastric juice (NaCl 2 g/L, pepsin 3.2 g/L, adjusted to pH 2.5 with concentrated HCl) and incubated at 37°C for 3 h. Solutions were centrifuged, the supernatant was discarded, and the pellets were suspended in MRS broth. Bacterial growth was evaluated by inoculating MRS broth with 2% v/v of control cells in saline and artificial gastric juice-treated cells onto microplates in triplicate, before incubating them in a Microplate Spectrophotometer System SpectraMax 340 (Molecular Devices Inc., Sunnyvale, CA, USA) at 37°C for 18 h. The OD_620nm_ (optic density) was recorded at 30 min intervals. The percentage of growth inhibition was calculated as (1 –areaAGJ/areaCT) x 100, where areaAGJ and areaCT are the areas under the growth curve for the simulated gastric juice and control, respectively. The total area under the curve was calculated by definite integration using the OriginPro 8.5 program (OriginLab Corporation, Northampton, MA, USA). The results were based on the average of three independent assays.

#### Susceptibility to bile salts

The susceptibility of *L*. *lactis* NCDO 2118 to bile salts was evaluated according to the method of Silva *et al*., (2013) [98]. For this, the *L*. *lactis* NCDO 2118 strain was grown in MRS medium at optical density of 0.6 and transferred (2% v/v) to MRS medium supplemented or not with 0.3% of Oxgall (Oxoid Ltd., Basingstoke, UK). The OD_620nm_ was recorded at 30 min intervals while incubating at 37°C for 18 h in a microplate reader. The percentage of growth inhibition was calculated as (1 –areaBS/areaCT) x 100, where areaBS and areaCT are the areas under the growth curve for bile salt and control cells, respectively. The percentage of bacterial viability was determined in a Microplate Spectrophotometer System SpectraMax 340 (Molecular Devices Inc., Sunnyvale, CA, USA) in the same manner as described above. The results were based on an average of three independent assays.

#### Cell surface hydrophobicity

MATS was measured to evaluate the bacterial cell surface hydrophobicity [99]. Measurement of the cell surface hydrophobicity of *L*. *lactis* NCDO 2118 was performed with xylene using the MATS method. Bacterial stationary phase cultures were centrifuged, washed twice and adjusted to an OD_600nm_ of 0.6 with 0.1 M KNO_3_, pH 6.2 (A_0_). Then, xylene was added in suspension 16% (v/v) and maintained for 10 minutes at room temperature. The tube was agitated vigorously, and after 30 minutes, the aqueous phase was collected for optical density OD_600nm_ measurement. The reduction percentage of optical density was calculated. The results were based on the average of three independent assays.

#### Antagonistic activity

Bacterial isolates were cultured in MRS broth for 24 h at 37°C within an anaerobic chamber. A 5 μL aliquot of the culture was then spotted onto MRS agar. After incubation at 37°C for 48 h under anaerobic conditions, the cells were killed by exposure to chloroform for 20 min. Residual chloroform was allowed to evaporate, and Petri dishes were overlaid with 3.5 mL of a soft agar containing brain heart infusion (Acumedia, Neogen Co., Lansing, MI, USA), tryptone soy broth (Difco) supplemented with 0.5% yeast extract (Acumedia), or Ellinghausen–McCullogh–Johnson–Harris with Leptospira enrichment EMJH (Difco) inoculated with 0.2 mL of a 24 h culture of *Staphylococcus aureus* ATCC 29213, *Enterococcus faecalis* ATCC 19433, *Pseudomonas aeruginosa* ATCC 25853, *Bacillus cereus* ATCC 11778, *Escherichia coli* ATCC 25723, *Salmonella enterica* serovar *Typhimurium* ATCC 14028, *Leptospira interrogans* serovar *Icterohaemorrhagiae*, or *Listeria monocytogenes* ATCC 15313. After incubating at 37°C for 24 h under aerobic or anaerobic conditions, depending on the indicator strain, the antagonistic activity was determined based on the presence of a growth inhibition zone, using the method of Tagg as modified by Branco *et al*., (2010) [100].

#### Antibiotic susceptibility

*L*. *lactis* NCDO 2118 antibiotic susceptibility was determined using antibiotic diffusion discs (Oxoid, England) on MRS plates. Bacteria were inoculated in MRS broth and incubated overnight at 37°C. Solutions of 10^8^ viable cells (McFarland scale) were prepared from the colonies in 3.5 mL of 0.9% buffered saline. The diluted culture (100 μL) was streaked onto MRS agar, and antibiotic discs were applied to the surface using an antibiotic disc dispenser. The discs included amikacin (30 μg), ampicillin (30 μg), ceftriaxone (30 μg), chloramphenicol (30 μg), erythromycin (10 μg), oxacillin (1 μg), penicillin G (10 U), tetracycline (30 μg) and vancomycin (30 μg). The results were interpreted according to Charteris *et al*., (1998) [101].

#### Bacterial strain, growth conditions and preparation of proteins from culture filtrates for proteomic analysis

*L*. *lactis* NCDO 2118 and *L*. *lactis* IL1403 were pre-inoculated in M17 medium (Difco, New Jersey, USA) and incubated at 30°C for 16 h. The precultures were then inoculated (1:100) in fresh M17 medium supplemented with 0.5% (w/v) glucose (M17Glc) at 30°C until reaching an OD_600_ = 0.8 (three independent experiments). The cultures were then centrifuged for 20 min at 2,700 x g. The supernatants were filtered using 0.22-μm filters, 30% (w/v) ammonium sulfate was added to the samples, and the pH of the mixtures was adjusted to 4.0. Next, 20 mL of N-butanol was added to each sample. The samples were centrifuged for 10 min at 1,350 x g and 4°C. The interfacial precipitate was collected and resuspended in 1 mL of 20 mM Tris-HCl pH 7.2 [102]. To perform label-free proteomic analysis, the protein extract was concentrated using a spin column with a 10 kDa threshold (Millipore, Billerica, MA, USA). The protein was denatured (0.1% *Rapi*GEST SF at 60°C for 15 min) (Waters, Milford, CA, USA), reduced (10 mM DTT), alkylated (10 mM iodoacetamide) and enzymatically digested with trypsin (Promega, Sequencing Grade Modified Trypsin, Madison, WI, USA).

#### Proteomic analysis

Qualitative and quantitative nanoUPLC tandem nanoESI-HDMS^E^ (Nano Electrospray High Definition Mass Spectrometry) experiments were performed using both a 1 h reversed phase gradient from 7% to 40% (v/v) acetonitrile (0.1% v/v formic acid) at 500 nL min^-1^ and a nanoACQUITY UPLC 2D RPxRP Technology system [103]. A nanoACQUITY UPLC HSS T3 1.8 μm, 75 μm × 15 cm column (pH 3) was used with an RP XBridge BEH130 C18 5 μm 300 μm x 50 mm nanoflow column (pH 10). Typical on-column sample loads were 250 ng of the total protein digests for each of the 5 fractions (250 ng/fraction/load). All analyses were performed using nano-electrospray ionization in the positive ion mode nanoESI (+) and a NanoLockSpray (Waters, Manchester, UK) ionization source. The mass spectrometer was calibrated using a MS/MS spectrum of [Glu^1^]-Fibrinopeptide B human (Glu-Fib) solution (100 fmol.μL^-1^) delivered through the NanoLockSpray source reference sprayer. The multiplexed data-independent (DIA) scanning with additional specificity and selectivity for non-linear ‘T-wave’ ion mobility (HDMS^E^) experiments were performed using a Synapt G2-S HDMS mass spectrometer (Waters, Manchester, UK).

Following the identification of proteins, the quantitative data were packaged using dedicated algorithms [104; 105] and searching against a database with default parameters to account for ions [106]. The databases used were reversed “on-the fly” during the database queries and appended to the original database to assess the false positive rate during identification. For proper spectra processing and database searching conditions, the ProteinLynxGlobalServer v.2.5.2 (PLGS) with Identity^E^ and Expression^E^ informatics v.2.5.2 (Waters) was used. UniProtKB with manually reviewed annotations was used, and the search conditions were based on taxonomy (*L*. *lactis*). The maximum allowed missed cleavages by trypsin were up to one, variable modifications by carbamidomethyl (C), acetyl N-terminal, phosphoryl (STY) and oxidation (M) were allowed, and a peptide mass tolerance value of 10 ppm was used [107]. The collected proteins were organized by the PLGS Expression^E^ tool algorithm into a statistically significant list that corresponded to higher or lower regulation ratios among the different groups. For protein quantification, the PLGS v2.5.2 software was used with the Identity^E^ algorithm using the Hi3 methodology. The search threshold to accept each spectrum was the default value in the program with a false positive value of 4%. The quantitative values were averaged over all samples, and the standard deviations at *p* < 0.05 were determined using the Expression software [107].

## Conclusions

Although *L*. *lactis* NCDO 2118 presented a high similarity to the other *L*. *lactis* strains, it presents an SI that is commonly shared with *L*. *lactis* KF147, along with high genomic synteny conservation with this strain. Additionally, the antibiotic resistance of this strain to vancomycin, amikacin and oxacillin could be an obstacle for its use as a probiotic. However, the absence of resistance-related genes in regions acquired by HGT and the absence of RIs in the genome sequence corroborates its safety aspects and supports its use as a probiotic strain. Moreover, the high susceptibility of *L*. *lactis* NCDO 2118 to acid and bile salts stresses have to be further evaluated in a complete digestion simulation, using transcriptomics and proteomics analyses, to elucidate whether the identified genes are differentially expressed in those environmental conditions.

Interestingly, the adhesion of *L*. *lactis* NCDO 2118 to xylene and the putative production of three classes of bacteriocins are important indicators of the exclusion mechanisms used by this strain. However, the *in vitro* analyses have not shown any sign of an antagonistic effect against the assayed pathogenic bacteria. Future works could also take advantage of combined transcriptomics and proteomics analyses of *L*. *lactis* NCDO 2118 *in vitro* before and after intestinal passage to evaluate the expression of the identified genes. Additionally, the identification of the EPS cluster of genes putatively associated with the probiotic effect of *L*. *lactis* NCDO 2118 could be further explored in 16S metagenomics analyses of gut microbiota, after expression, purification and administration of EPS proteins. Finally, through the analyses of the safety, survival and probiotic aspects of *L*. *lactis* NCDO 2118, we highlight here the potential use of this strain as a target for the future development of probiotic foods.

## Supporting information

S1 Fig Gene synteny between *Lactococcus lactis* subsp. *lactis* strains. *L*. *lactis* subsp. *lactis* NCDO 2118 (top) was used as a reference for the comparison analyses. The genomes are represented according to the nucleotide conservation and synteny. Low similarity regions are represented as white regions inside the blocks, highlighted by a red (*). Regions of deletions are represented as blank spaces between the blocks, letter (A). Insertion regions are highlighted with the letter (B), and inversion regions are represented by the letter (C). To perform the genome synteny analysis, we used the software Mauve, which compares the genomes by identifying and clustering homologous genes between the genomes into large collinear blocks of genes [89]. The most conserved genome compared to *L*. *lactis* NCDO 2118 was *L*. *lactis* KF147. Between these two strains, it is possible to see some regions of: deletion; insertion; inversion and specific areas with low or no similarity with the reference genome. The comparison of those features with other strains shows: a deletion on the genome position 1,200,000 of *Lactococcus lactis* subsp. *lactis* IO-1; a big inversion region in *Lactococcus lactis* subsp. *lactis* AI06 in the range from 800,000 to 1,600,000; a small insertion near the genome position 200,000 of *L*. *lactis* KLDS 40325 (in green); and a block on *Lactococcus lactis* subsp. *lactis* S0 (2,000,000 position) with low similarity to the reference genome.
(TIF)

S2 Fig Growth curves of *L*. *lactis* NCDO 2118 under acid and bile salt stresses. (A) *L*. *lactis* subsp. *lactis* NCDO 2118 growth under acid stress conditions. Blue: (LL) *L*. *lactis* without acid contact. Red: (LLAT) *L*. *lactis* under acid treatment. (B) *L*. *lactis* growth under intestinal conditions. Blue: (LL) *L*. *lactis* without salt contact salt. Red: (LLOG) *L*. *lactis* growth with 0.3% ox gall.
(TIF)

S1 Table Metabolic pathways exclusive of *Lactococcus lactis* subsp. *lactis* NCDO 2118. The metabolic pathways were predicted using the software Pathway Tools.
(XLS)

S2 Table Putative genomic islands of *L*. *lactis* subsp. *lactis* NCDO 2118. (XLSX)

S3 Table Genes coding for bacteriocins, muramidases and macrolides. Bacteriocin regions were predicted using BAGEL.
(XLS)

S4 Table Exclusive, expressed and secreted proteins of *L*. *lactis* NCDO 2118. The exclusive, secreted and expressed proteins were predicted using the software OrthoMCL, SurfG+ and proteomics analyses, respectively.
(XLS)
