# Supplementary figures and images for: Analyses of the probiotic property and stress resistance-related genes of Lactococcus lactis subsp. lactis NCDO 2118 through comparative genomics and in vitro assays
Source: PLoS One. 2017 Apr 6;12(4):e0175116. doi: 10.1371/journal.pone.0175116 (PMC5383145; doi:10.1371/journal.pone.0175116)

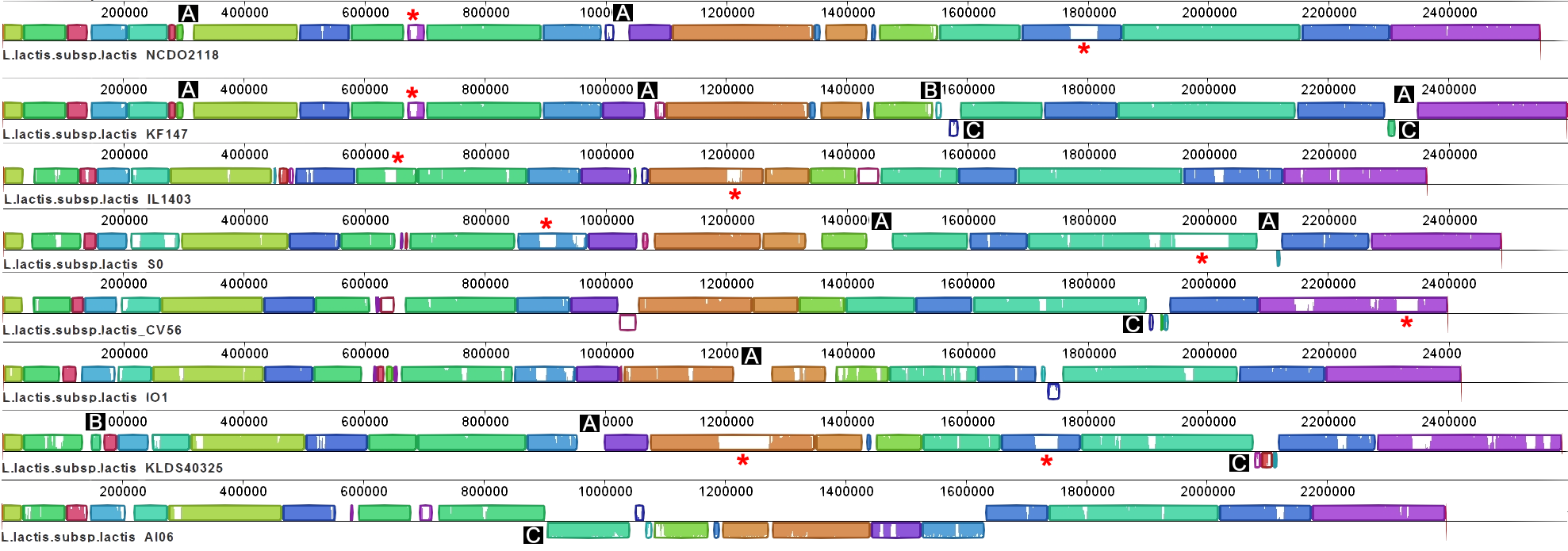

Supplement: S1 Fig — L. lactis subsp. lactis NCDO 2118 (top) was used as a reference for the comparison analyses. The genomes are represented according to the nucleotide conservation and synteny. Low similarity regions are represented as white regions inside the blocks, highlighted by a red (*). Regions of deletions are represented as blank spaces between the blocks, letter (A). Insertion regions are highlighted with the letter (B), and inversion regions are represented by the letter (C). To perform the genome synteny analysis, we used the software Mauve, which compares the genomes by identifying and clustering homologous genes between the genomes into large collinear blocks of genes [89]. The most conserved genome compared to L. lactis NCDO 2118 was L. lactis KF147. Between these two strains, it is possible to see some regions of: deletion; insertion; inversion and specific areas with low or no similarity with the reference genome. The comparison of those features with other strains shows: a deletion on the genome position 1,200,000 of Lactococcus lactis subsp. lactis IO-1; a big inversion region in Lactococcus lactis subsp. lactis AI06 in the range from 800,000 to 1,600,000; a small insertion near the genome position 200,000 of L. lactis KLDS 40325 (in green); and a block on Lactococcus lactis subsp. lactis S0 (2,000,000 position) with low similarity to the reference genome. (TIF) [file pone.0175116.s001.tif]

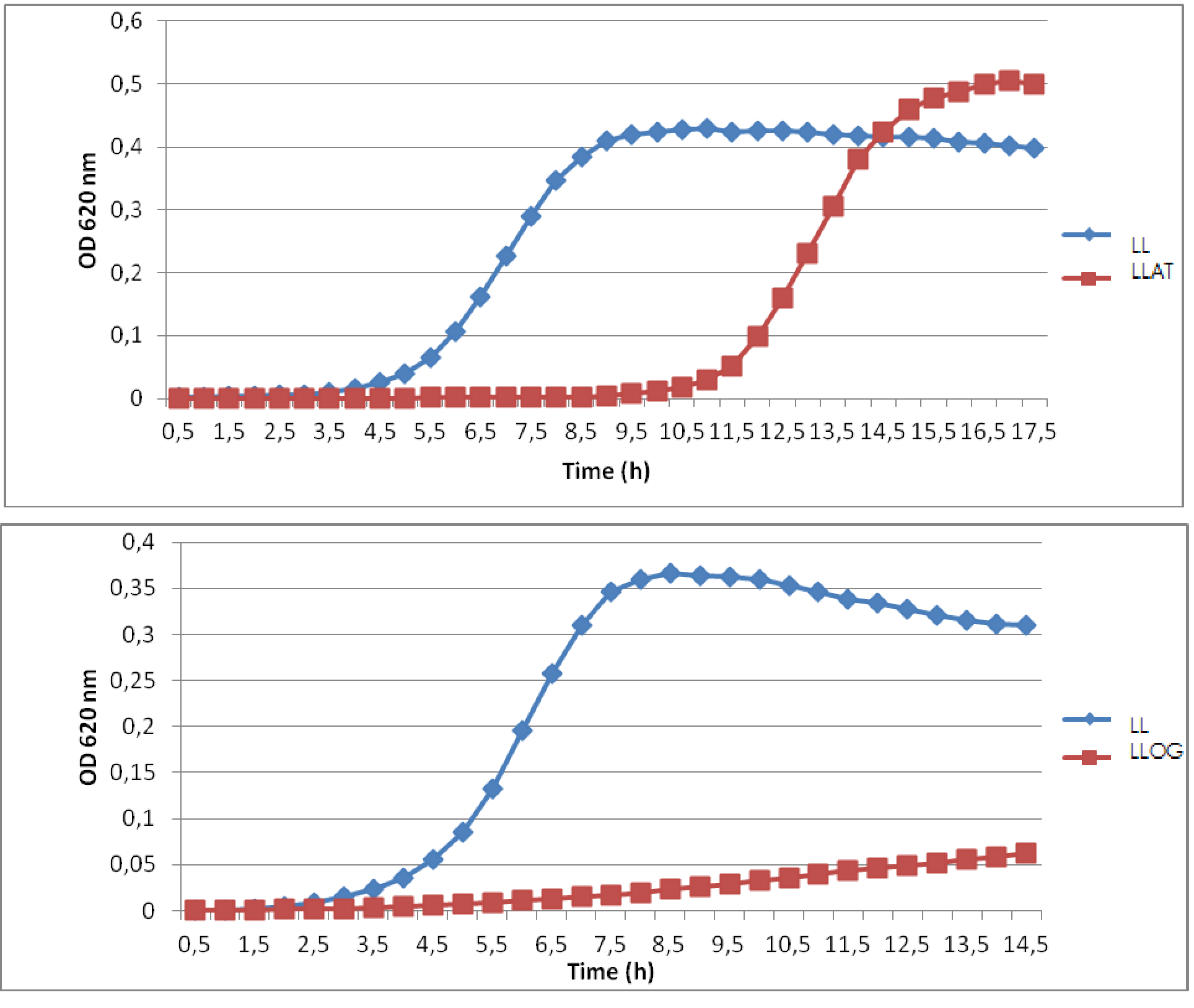

Supplement: S2 Fig — (A) L. lactis subsp. lactis NCDO 2118 growth under acid stress conditions. Blue: (LL) L. lactis without acid contact. Red: (LLAT) L. lactis under acid treatment. (B) L. lactis growth under intestinal conditions. Blue: (LL) L. lactis without salt contact salt. Red: (LLOG) L. lactis growth with 0.3% ox gall. (TIF) [file pone.0175116.s002.tif]
